# Novel reverse electrodialysis-driven iontophoretic system for topical and transdermal delivery of poorly permeable therapeutic agents

**DOI:** 10.1080/10717544.2017.1367975

**Published:** 2017-08-28

**Authors:** Ki-Taek Kim, Joon Lee, Min-Hwan Kim, Ju-Hwan Park, Jae-Young Lee, Joo-Hyun Song, Minwoong Jung, Myoung-Hoon Jang, Hyun-Jong Cho, In-Soo Yoon, Dae-Duk Kim

**Affiliations:** a College of Pharmacy and Research Institute of Pharmaceutical Sciences, Seoul National University, Gwanak-gu, Seoul, Republic of Korea;; b Biosensor Laboratories Inc, Seoul National University, Gwanak-gu, Seoul, Republic of Korea;; c School of Chemical and Biological Engineering, Seoul National University, Gwanak-gu, Seoul, Republic of Korea;; d College of Pharmacy, Chungnam National University, Yuseong-gu, Daejeon, Republic of Korea;; e College of Pharmacy, Kangwon National University, Chuncheon-si, Gangwon, Republic of Korea;; f College of Pharmacy, Pusan National University, Geumjeong-gu, Busan, Republic of Korea

**Keywords:** Reverse electrodialysis, iontophoresis, transdermal delivery, topical delivery, vitamin C, hyaluronic acid, lopinavir

## Abstract

Topical and transdermal drug delivery has great potential in non-invasive and non-oral administration of poorly bioavailable therapeutic agents. However, due to the barrier function of the stratum corneum, the drugs that can be clinically feasible candidates for topical and transdermal delivery have been limited to small-sized lipophilic molecules. Previously, we fabricated a novel iontophoretic system using reverse electrodialysis (RED) technology (RED system). However, no study has demonstrated its utility in topical and/or transdermal delivery of poorly permeable therapeutic agents. In this study, we report the topical delivery of fluorescein isothiocyanate (FITC)–hyaluronic acid (FITC–HA) and vitamin C and the transdermal delivery of lopinavir using our newly developed RED system in the *in vitro* hairless mouse skin and *in vivo* Sprague–Dawley rat models. The RED system significantly enhanced the efficiency of topical HA and vitamin C and transdermal lopinavir delivery. Moreover, the efficiency and safety of transdermal delivery using the RED system were comparable with those of a commercial ketoprofen patch formulation. Thus, the RED system can be a potential topical and transdermal delivery system for various poorly bioavailable pharmaceuticals including HA, vitamin C, and lopinavir.

## Introduction

Topical and transdermal drug delivery can offer great potential for non-invasive and non-oral administration of poorly bioavailable therapeutic agents (Kalia et al., 2004; Chen et al., 2016; Jacobs et al., 2016; Zidan et al., 2016). Drug delivery through the skin has merits in improving patient compliance and avoiding gastrointestinal irritation and hepatic first-pass effect (Baek & Cho, 2016; Jain et al., 2017). Moreover, topical delivery can enhance the efficiency of drug targeting to the skin and adjacent tissues, while transdermal delivery can provide a constant systemic exposure profile, potentially reducing several side effects caused by fluctuating blood drug concentrations (Jung et al., 2015; Patel et al., 2016; Rao et al., 2016). The outermost layer of the skin, i.e. the stratum corneum, has a multilamellar lipid structure reinforced with a dense network of keratin, which protect internal tissues against desiccation, infection, xenobiotic chemicals, and mechanical stress (Rawlings & Harding, 2004; Sadowski et al., 2017). However, owing to its barrier function, the drugs that can be clinically feasible candidates for topical and transdermal delivery have been severely limited to highly potent lipophilic molecules with low molecular weight (<500 Da) (Naik et al., 2000; Prausnitz et al., 2004; Hussain et al., 2016).

Among various delivery systems that can enhance drug penetration across the skin, iontophoretic systems have attracted great interest as a useful tool for hydrophilic and/or large-sized molecules (Banga, 2007; Sachdeva et al., 2010; Saluja et al., 2013). It is a non-invasive technique to drive a charged substance through the skin by a low level of current (typically <500 μA/cm^2^) generated from a small electrical potential (Calatayud-Pascual et al., 2011). However, the conventional iontophoresis-based transdermal delivery systems require a circuit system connected to an external power supply that is generally non-portable, non-disposable, hazardous, and expensive (Calatayud-Pascual et al., 2011; Vikelis et al., 2012; Saluja et al., 2013).

**Figure 1.**
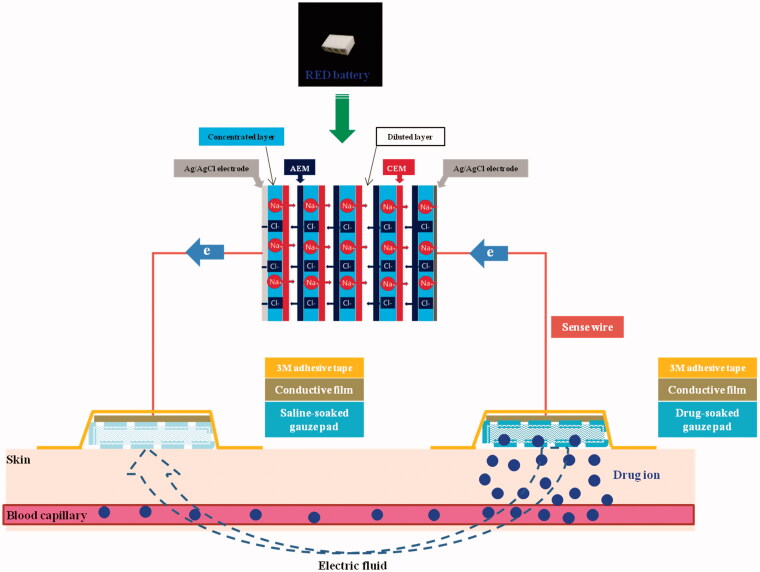
Schematic diagram of RED system.

To remedy the disadvantage of conventional iontophoretic systems, we recently developed a novel iontophoretic system using reverse electrodialysis (RED) technology [RED-driven iontophoretic system (RED system); Figure 1] (Lee et al., 2017). This technology uses the salinity gradient energy produced by the controlled mixing of salt water (brine) and fresh water through alternating ion exchange membranes, which generates chemical potential difference over each membrane (Długołęcki et al., 2008; Post et al., 2008; Veerman et al., 2009). The electric current-generating part of the RED system (RED battery) was fabricated by stacking a series of cells containing a pair of anion- and cation-exchange membranes and placing them between two electrodes (Lee et al., 2017). The electric potential of the RED battery is the sum of the potential difference generated from all the membranes, which can be readily controlled by adjusting the size and numbers of the stacked cells. Moreover, the RED battery is disposable and eco-friendly because it uses only brine and water as fuel (Lee et al., 2017).

In our previous study, we evaluated the electrical performance of the RED system and its enhancing effect on the permeation of fluorescein isothiocyanate (FITC)-poly-l-lysine (FITC-PLL) through the porcine skin (Lee et al., 2017). However, until date, no study has demonstrated the utility of the RED system in the topical and/or transdermal delivery of therapeutic agents whose dermal applications in clinical settings have been limited due to their poor skin permeability.

Hyaluronic acid (HA) is an anionic non-sulfated glycosaminoglycan comprising repeating polymeric disaccharides of glucuronic acid and acetylglucosamine (Mandal et al., 2016). It is an anti-wrinkle and moisturizing agent widely used in the area of pharmaceutics and cosmetics. It also plays pivotal roles in regulating the response of dermal fibroblasts and epithelial cells to skin injury and activating inflammatory cells to stimulate immune response during extracellular matrix synthesis and wound healing (Papakonstantinou et al., 2012). Moreover, it has been reported that the level of HA in the epidermis/dermis decreases to five percent of baseline with aging (Jegasothy et al., 2014). Vitamin C, a well-known antioxidant agent, can protect the skin from UV-induced reactive oxygen species and regulate collagen synthesis in the epidermis/dermis. It inhibits the action of tyrosinase which is the key enzyme in melanogenesis (Sauermann et al., 2004). Similar to HA, the level of vitamin C in the epidermis tends to decrease with aging (Leveque et al., 2002). Thus, delivering HA and vitamin C to the epidermis/dermis is a crucial prerequisite for exerting their therapeutic efficacy in skin health care. Lopinavir, a human immunodeficiency virus (HIV) protease inhibitor, has a low and variable oral bioavailability due to extensive intestinal and hepatic first-pass metabolism mediated by cytochrome P450 (CYP) 3A4 (ter Heine et al., 2011). To overcome the poor bioavailability, lopinavir is currently co-administered with low-dose ritonavir, a potent CYP3A4 inhibitor, for enhancing the systemic exposure to lopinavir. However, the use of bioavailability boosters in antiretroviral therapy may result in adverse drug–drug interaction and toxicity in HIV patients (Kumar et al., 2015). As transdermal drug administration can enhance and prolong systemic exposure compared with oral administration (Jung et al., 2015), the development of transdermal delivery systems of lopinavir is highly warranted to improve the efficacy and safety of lopinavir-based antiretroviral therapy. However, in clinical settings, the topical or transdermal delivery of the above-mentioned three therapeutic agents has been severely limited due to their large molecular size (about 10,000 Da for HA and 628.8 Da for lopinavir) and/or low lipophilicity (Log *P* = −1.8 for HA and −1.85 for vitamin C). Therefore, we selected FITC-HA, vitamin C, and lopinavir to demonstrate the feasibility of our RED system as an effective topical and transdermal drug delivery system for poorly permeable therapeutic agents.

Herein, we report the topical delivery of FITC-HA and vitamin C and the transdermal delivery of lopinavir using the RED system in the *in vitro* hairless mouse skin and *in vivo* Sprague–Dawley rat models. In addition, the transdermal delivery efficiency and safety of the RED system were compared with those of the Ketotop® patch, a commercial transdermal patch formulation of ketoprofen (Oh et al., 2017).

## Materials and methods

### Materials

FITC-PLL (FITC-linker-K-K-K-K-K; purity >93%; 1,160 Da) was purchased from Peptron Inc. (Daejeon, Korea). FITC-HA (purity >95%; 10,000 Da) was purchased from Creative PEGWorks Co. (Chapel hill, NC). Vitamin C, ketoprofen, and sodium lauryl sulfate (SLS) were purchased from Sigma-Aldrich Chemical Co. (St. Louis, MO). Ketotop^®^ patch was purchased from Handok Pharmaceutical Co., Ltd. (Seoul, Korea). Lopinavir was purchased from Toronto Research Chemicals Inc. (Toronto, Ontario, Canada). Valsartan was purchased from Tokyo Chemical Industry Co., Ltd. (Tokyo, Japan). All other reagents were of analytical grade and used without further purification

### Preparation and characterization of RED system

The RED system was fabricated by our previously reported method with slight modifications (Lee et al., 2017). In brief, the RED system used in this study comprised RED battery, sense wire (Keithley Instruments Inc., Cleveland, OH), conductive carbon film (Suzhou HC Plastic Co. Ltd., Anhui, China), and gauze pad (for drug loading; KM Supplies Inc., Los Angeles, CA) (Figure 1). The RED battery comprised electrodes, non-woven fabrics, cation exchange membranes (CEMs), and anion exchange membranes (AEMs). Homogeneous Selemion^TM^ CMV and AMV membranes (Asahi Glass CO. Ltd., Tokyo, Japan) were used as the CEMs and AEMs, respectively. A non-woven fabric (SP60, NamYang Nonwoven Fabric CO. Ltd., Ansan, Korea) with 0.3-mm thickness was used in both the concentrated and diluted layers. Sodium chloride (NaCl) solution was added to the concentrated layer and dried. The Ag/AgCl electrode was fabricated by an electroplating method. The bottom and lateral sides of the RED battery were tightly sealed with a typical epoxy to avoid any leaks. The upper side of the RED battery was kept open to allow the supply of diluted NaCl solution. The active area for ion exchange in eight cell pairs was 2.4 cm × 0.6 cm. To confirm the generation of electric current from the RED system, a bulb lighting test was performed (see the Supplementary Information, Video S1 and Figure S1).

### Animals

Male hairless mice (6–8 weeks of age) and male Sprague–Dawley (SD) rats (5 weeks of age) were used for the *in vitro* and *in vivo* studies, respectively. They were purchased from OrientBio Inc. (Seongnam, Korea) and maintained in a clean room (Animal Center for Pharmaceutical Research, College of Pharmacy, Seoul National University) at a temperature of 20–23 °C with a 12/12-h light (7 am–7 pm)/dark (7 pm–7 am) cycle and a relative humidity of 50 ± 5%. The animals were housed in metabolic cages (Tecniplast USA Inc., West Chester, PA) under filtered and pathogen-free air, with food (Agribrands Purina Canada Inc., Levis, QC, Canada) and water available *ad libitum*. Experimental protocols for the current animal studies were reviewed by the Animal Care and Use Committee of the College of Pharmacy, Seoul National University (Mouse study: SNU-111007-4-2; Rat study: SNU-160311-3-1), in accordance with the National Institutes of Health’s Guide for the Care and Use of Laboratory Animals (National Institutes of Health Publication Number 85-23, revised 1985).

### Application of RED system for *in vitro* studies

Keshary–Chien diffusion cells with a surface area of 4.41 cm^2^ at 32 °C were used for *in vitro* skin deposition and permeation studies. After sacrificing hairless mice by cervical dislocation, the dorsal skin was cut to about 5 cm × 5 cm and the subcutaneous fat was removed. Then, the skin was fixed tightly between the donor and receptor cells, laying the stratum corneum toward the donor cells. A gauze pad soaked with drug solution was placed on the stratum corneum side of skin (donor cells). Then, the conductive carbon film was mounted on the gauze pad and tightly fixed on the skin with 3 M adhesive tape. The edge of the film was connected to one of the two electrodes (either the anode or cathode) of the RED battery using sense wire. The other electrode of the RED battery was extended with Ag/AgCl wire and dipped into the receptor cells which were filled with 22.0-mL PBS (pH 7.4; consisting of 1.1 mM KH_2_PO_4_, 5.6 mM NaHPO_4_, and 155 mM NaCl) and continuously stirred with magnetic bar (600 rpm). The RED-driven iontophoretic reaction was initiated by adding NaCl solution to the upper side of the RED battery. The images of the set up of RED system applied to the diffusion cells for the *in vitro* skin deposition and permeation study are provided in the Supplementary Information (Figure S2A).

### CLSM analysis

The CLSM analysis was performed as previously described (Wang et al., 2011). The FITC-PLL or FITC-HA-loaded RED system (RED) was applied on the stratum corneum side of the skin fixed in the Keshary–Chien diffusion cells as described above. Gauze pad of donor side was soaked with FITC-PLL (0.116 mg) or FITC-HA (0.2 mg) dissolved in PBS. In the RED system, the FITC-PLL or FITC-HA-loaded part was connected to the cathode of the RED battery. For comparison, the FITC-PLL or FITC-HA-soaked gauze pad without the RED system (control) was applied to the other diffusion cells. At 1 h and 6 h after the application of the control and RED systems, the skin was removed from the diffusion cells, washed with PBS, cut to 1 cm × 1 cm, and placed on a glass slide, laying the stratum corneum side faced downward. A CLSM (TCS SP8 X; Leica Microsystems, Ltd., Wetzlar, Germany) was employed to visualize the distribution of FITC-PLL and FITC-HA applied on the hairless mouse skin. The system was equipped with an white-light laser source whose fluorescence emission wavelength was 530 nm. Z-stacks profiles of the prepared skin samples were generated by capturing individual images from the surface of the skin to the interior at an interval of 3 µm. A fluorescent signal emitted from FITC-PLL or FITC-HA was represented by green color. The intensity of the fluorescent signal was quantified using LAS-AF-Lite software program (Version 2.6.3; Leica Microsystems, Ltd., Wetzlar, Germany).

### *In vitro* hairless mouse skin deposition study

For the *in vitro* skin deposition study of vitamin C, gauze pad of donor side was soaked with vitamin C (12.5 mg) dissolved in PBS and applied on the stratum corneum side of skin fixed in the diffusion cell as described above. The vitamin C-loaded part was connected to the cathode of the RED battery for the RED system group, while the control group was not. At 6 h after the application of the control and RED systems, the skin was removed from the diffusion cells and washed five times with 50% methanol (5 mL each time). The washing procedure was validated and found to remove >95% of the initial dose applied on the skin surface at time zero (Singh et al., 2010). The amount of vitamin C deposited in the stratum corneum was determined by a tape stripping method (Gopee et al., 2009; Lademann et al., 2009; Rissmann et al., 2009; Aripin et al., 2013). In brief, a cellophane adhesive tape (CuDerm Co., Dallas, TX) was pressed on the skin surface ten times with a roller of 1 kg in weight (the weight of the roller itself was used to press the tape) and then pulled off with one fluent stroke. This tape striping procedure was repeated 10 times using one tape at each repetition. The tape samples were extracted with 3 mL of 50% methanol for 3 h and centrifuged at 16,100 × *g* for 5 min. The skin samples whose stratum corneum was removed (epidermis and dermis) were chopped, collected into a 15-mL tube containing 3 mL of 50% methanol, homogenized using ULTRA-TURAX^®^ T25basic (IKA, Staufen, Germany), and centrifuged at 16,100 × *g* for 5 min. The amount of vitamin C in the supernatant was determined by LC-MS/MS analysis.

### *In vitro* hairless mouse skin permeation study

The *in vitro* skin permeation studies of lopinavir and ketoprofen were performed as described above. For lopinavir, 6.5 mg of lopinavir dissolved in 100% propylene glycol was loaded in gauze pad, and it was connected to the anode of the RED battery, while the control group was not. For ketoprofen, 3.86 mg of ketoprofen dissolved in 50% propylene glycol was loaded in gauze pad, and it was connected to the cathode of the RED battery, while the control group was not. In addition, the commercial Ketotop^®^ patch (3.86 mg as ketoprofen) without the RED system was applied in the same manner as the control group. At 1, 2, 3, 4, 5, 6, 7, 8, 9, and 10 h after the application, 0.5-mL aliquots were taken from the receptor cell and replaced immediately with an equal volume of fresh receptor medium. The amount of lopinavir and ketoprofen in the samples was determined by LC-MS/MS analysis.

### *In vivo* pharmacokinetic study in rats

The abdominal hair of rats was removed using an electric clipper and depilatory cream. After 1 d, the rats were anesthetized with intramuscular injection of zoletil (Virbac Korea Co. Ltd., Seoul, Korea), and their femoral artery was cannulated with a polyethylene tube (PE-50; Clay Adams, Parsippany, NJ) filled with heparinized saline (20 IU/mL) to prevent blood clotting (Heo et al., 2016; Kim et al., 2017). Then, two gauze pads (one soaked with drug solution and other with saline) were placed on the shaven abdominal skin spaced slightly apart. Then, the conductive carbon film was mounted on each of the two gauze pads and they were tightly fixed on the skin with 3 M adhesive tape. The edge of each of the two films was connected to each of the two electrodes of the RED battery using sense wire. The RED-driven iontophoretic reaction was initiated in the same manner as that in the *in vitro* study. The images of the rats fixed on a plate for *in vivo* rat studies using the RED system are provided in the Supplementary Information (Figure S2B). The same experimental conditions as those tested in the *in vitro* skin permeation studies of lopinavir (control and RED) and ketoprofen (control, RED, and Ketotop^®^) were applied on the shaven abdominal skin of rats as described above. About 200-μL aliquots of blood were collected from the femoral artery at 0.5, 1, 2, 3, 4, 6, 9, 12, and 24 h after the transdermal application. Plasma samples were obtained by centrifugation at 16,100 × *g* for 5 min and stored at −80 °C until LC-MS/MS analysis.

### *In vivo* dermal toxicity study in rats

To assess the skin irritation caused by the RED system, the rat skin erythema test was performed using a spectrophotometer (CM-2500d; Konica Minolta Inc., Tokyo, Japan) as previously described (Banks et al., 2010; Kadam & Chuan, 2016). In brief, the abdominal hair of rats was removed using an electric clipper and depilatory cream. Saline solution, saline-soaked gauze dressing without the RED system, and the same experimental group as those tested in the *in vitro* skin permeation studies of ketoprofen (control, RED, and Ketotop^®^) were applied on the shaven abdominal skin of rats as described above. After 1 and 24 h, the tested formulations were removed carefully from the skin, and the skin erythema were measured using the spectrophotometer, which projects white light onto the skin and measures the color of light reflected from the skin. The erythema index was calculated from the following equation:

(1) Erythema index=100×log (Ir/Ig)

where Ir is the intensity of red component such as hemoglobin (R value) of reflected light and Ig is the intensity of green component such as bilirubin (G value) of reflected light.

### LC-MS/MS analysis of vitamin C, lopinavir, and ketoprofen

The amount of vitamin C in mouse skin homogenate samples was determined by LC-MS/MS analysis. The samples were diluted adequately with mobile phase solution [acetonitrile: DW containing 0.2% formic acid (70:30, v/v)] and transferred into the MS vials and a 3-µL aliquot was injected into Agilent LC-MS/MS system (Agilent Technologies, Palo Alto, CA) through Poroshell EC C18 column (50 mm × 3.0 mm, 2.6 µm; Agilent Technologies, Palo Alto, CA). MS detection was operated in the multiple reaction monitoring (MRM) mode with negative electrospray ionization (ESI). The isocratic mobile phase was run at a flow rate of 0.3 mL/min. The retention time of vitamin C was 1.7 min. The signal-to-noise ratio on LLOQ (100 ng/mL) was higher than 5.0 and there was no interference around the peak of vitamin C (Figure S3). The calibration standard samples were prepared with a final concentration of 100–5000 ng/mL. The calibration curves for lopinavir were linear with the *r*^2^ of more than 0.999.

The concentrations of lopinavir and ketoprofen in buffer solution used in the receptor cells (buffer samples) and rat plasma samples were determined by LC-MS/MS analysis. A 3-µL aliquot of the pretreated samples was injected into an Agilent LC-MS/MS system (Agilent Technologies, Palo Alto, CA) operated in the multiple reaction monitoring (MRM) mode with positive electrospray ionization (ESI). For the analysis of lopinavir, the samples were injected onto Poroshell EC C18 column (50 mm × 3.0 mm, 2.6 µm; Agilent Technologies, Palo Alto, CA). The isocratic mobile phase composed of acetonitrile and DW containing 0.2% formic acid (75:25, v/v) was run at a flow rate of 0.4 mL/min. The retention times of lopinavir and IS were 2.6 and 2.1 min, respectively. The signal-to-noise ratio on LLOQ (5.0 ng/mL) was higher than 5.0 and there was no interference around the peak of lopinavir. The calibration standard samples were prepared with a final concentration of 5.0–1000 ng/mL. The calibration curves for lopinavir were linear with the *r*^2^ of more than 0.999.

For the analysis of ketoprofen, the prepared samples were injected onto Hypersil BDS C18 column (50 mm × 4.6 mm, 5 µm; Thermo Fisher Scientific Co., Waltham, MA). The isocratic mobile phase, composed of acetonitrile and DW containing 0.2% formic acid (30:70, v/v), was run at a flow rate of 0.3 mL/min. The retention times of ketoprofen and IS were 0.80 and 0.77 min, respectively. The signal-to-noise ratio on LLOQ (20.0 ng/mL) was higher than 5.0 and there was no interference around the peak of ketoprofen. The calibration standard samples were prepared with a final concentration of 20.0–5000 ng/mL. The calibration curves for ketoprofen were linear with the *r*^2^ of more than 0.999. The analytical data were processed using the MassHunter Workstation Software Quantitative Analysis (vB.05.00; Agilent Technologies, Palo Alto, CA). The analytical conditions used for the mass detection of the analytes are summarized in Table 1.

**Table 1.** Mass detector parameters used for the analysis of vitamin C, lopinavir, ketoprofen, and valsartan (IS).

	Analytes			
Parameter	Vitamin C	Lopinavir	Ketoprofen	IS
*m*/*z* value of precursor ion	175.0	629.7	255.1	436.2
*m*/*z* value of product ion	114.9	447.3	209.1	291.2
Fragment voltage (V)	113	126	110	98
Collision energy (eV)	10	12	11	14
Cell accelerator voltage (V)	1	1	1	5
Gas temperature (°C)	300	300	350	–^a^
Gas flow (L/min)	9	11	11	–
Nebulizer pressure (psi)	25	30	15	–
Capillary voltage (V)	5000	3000	5000	–

^a^ Same as lopinavir or ketoprofen.

### Data analysis

*In vitro* skin permeation parameters were calculated by plotting the cumulative amount of drug permeated through the skin per unit area *versus* time. The slope of the linear portion in the plot was calculated as the flux (μg/h/cm^2^) (Ibrahim et al., 2015; Salah et al., 2017). The lag time was calculated by extrapolating the linear portion to the X-axis of the plot (Rao et al., 2015; Salah et al., 2017). Non-compartmental analysis was performed using the WinNonlin software (Version 4.0; Certara USA Inc., Princeton, NJ) to determine the *in vivo* pharmacokinetic parameters. The peak drug concentration in plasma (*C*_max_) and time to reach *C*_max_ (*T*_max_) were read directly from the observed data points. The area under the plasma drug concentration *versus* time curve from time 0 to 24 h (AUC_last_) was calculated by the linear trapezoidal method. The drug concentration at steady state (*C*_ss_) was calculated by dividing the AUC_last_ by time (24 h).

### Statistical analysis

All data were expressed as means ± standard deviation. A *p* value of less than .05 was considered to be statistically significant using a two-tailed unpaired Student’s *t*-test or one-way ANOVA test with Tukey’s multiple comparison.

## Results

### Effects of RED system on skin distribution of FITC-PLL and FITC-HA

To assess the enhancement of skin permeation by the RED system, the skin distribution of FITC-PLL and FITC-HA was visualized by confocal laser scanning microscopy (CLSM) with or without the application of RED system. Figures 2(A), S5, and S6 show the Z-stack, *X*/*Z* penetration, and *X*/*Y*/*Z* penetration CLSM images of hairless mouse skin at 1 h and 6 h after the application of FITC-PLL- or FITC-HA-loaded control or RED system. The Z-stack images at a skin depth of 3 μm for FITC-PLL and FITC-HA, respectively, were representative of the stratum corneum, while those at 12 μm were representative of the epidermis/dermis (Figure 2(A)). The fluorescence intensities at the stratum corneum and epidermis/dermis skin depth have been summarized in Figure 2(B). As shown in Figure 2(A), no background green fluorescence was observed in the intact skin. Overall, the fluorescence density of FITC-PLL and FITC HA tended to be higher at 6 h than at 1 h as well as markedly higher in the FITC-PLL-loaded groups than in the FITC-HA-loaded groups. Furthermore, the CLSM images at various skin depths demonstrated that both FITC-PLL and FITC-HA were absorbed deeper into the skin following the RED system application and FITC-PLL showed stronger fluorescence intensity than FITC-HA (Figures S5 and S6). As shown in Figure 2(B), for FITC-PLL, no significant difference in fluorescence density values at the stratum corneum was observed between the control and RED groups. However, the fluorescence density value at the epidermis/dermis was significantly higher in the RED group than in the control group. For FITC-HA, the fluorescence density values at the stratum corneum and epidermis/dermis were significantly higher in the RED group than in the control group.

Figure 2.Z-stack CLSM images (A) and fluorescence intensities (B) at the stratum corneum and epidermis/dermis of hairless mouse skin at 1 h and 6 h after the application of FITC-PLL- or FITC-HA-soaked gauze dressing without the RED system (control) and FITC-PLL- or FITC-HA-loaded RED system (RED) on the mouse skin fixed in the diffusion cells. A fluorescent signal emitted from FITC-PLL or FITC-HA was represented by green color. The scale bars represent 100 μm. The rectangular bars and their error bars represent the means and standard deviations (*n* = 3–4). The asterisk (*) represents a value significantly different from that of the intact skin group, and the pound sign (#) represents a value significantly different from that of the control group (*p* < .05).

**Figure:**
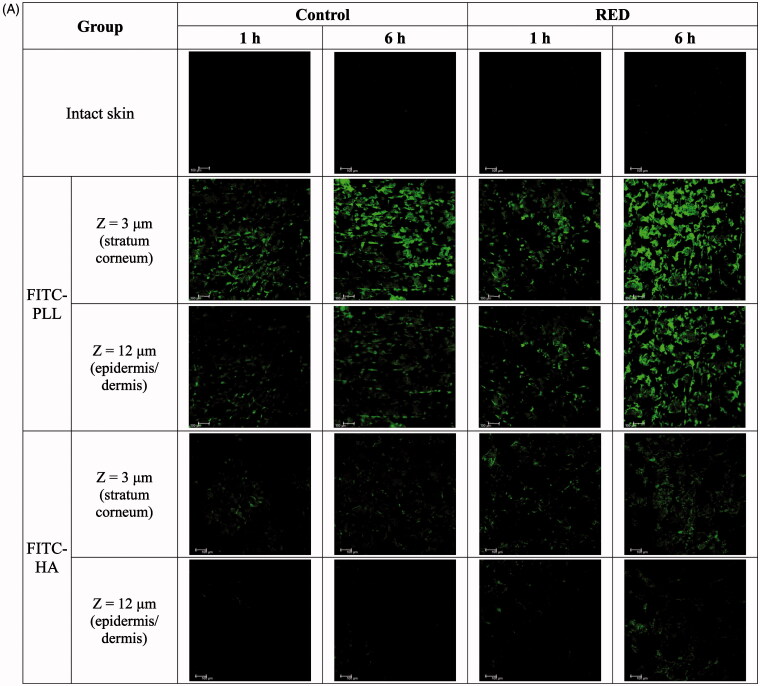

**Figure:**
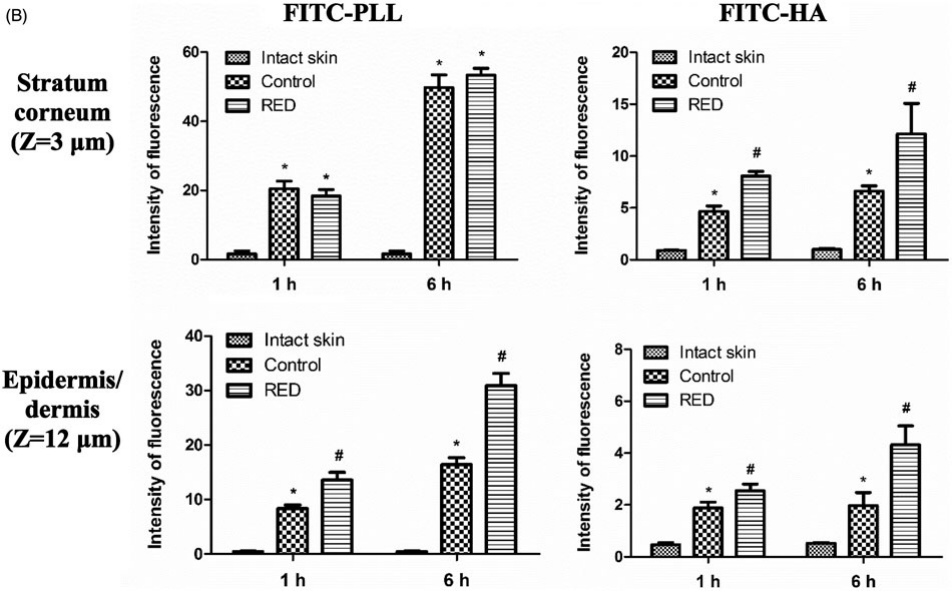

### Effects of RED system on *in vitro* skin deposition of vitamin C

**Figure 3.**
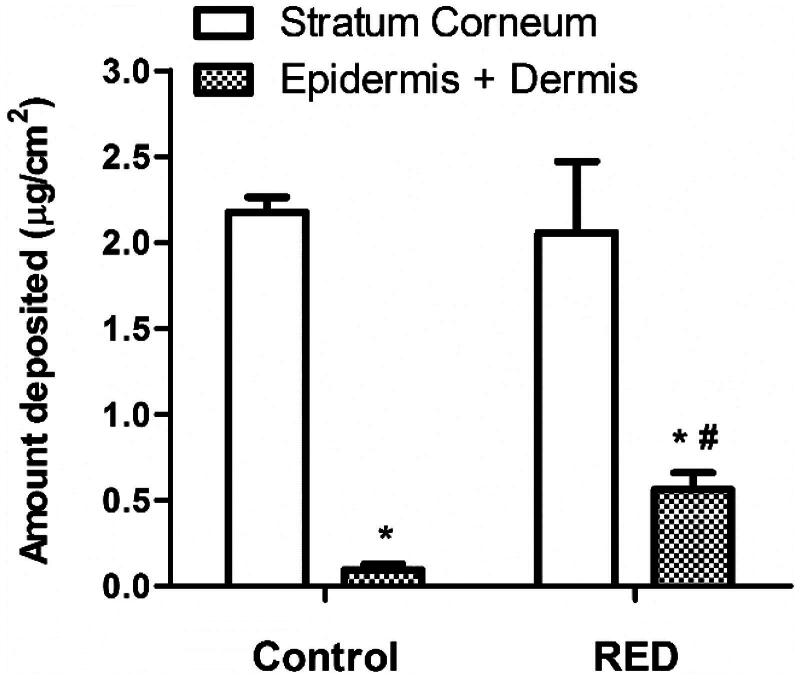
The amount of vitamin C deposited in the stratum corneum and epidermis/dermis of hairless mouse skin at 6 h after the application of vitamin C-soaked gauze dressing without the RED system (control) and vitamin C-loaded RED system (RED) on the mouse skin fixed in the diffusion cells. The rectangular bars and their error bars represent the means and standard deviations (*n* = 3–4). The asterisk (*) represents a value significantly different from that of the stratum corneum, and the pound sign (#) represents a value significantly different from that of the control group (*p* < .05).

The effects of RED system on the skin deposition of vitamin C, an antioxidant agent, were investigated. Figure 3 shows the amount of vitamin C deposited in the stratum corneum and epidermis/dermis of hairless mouse skin at 6 h after the application of vitamin C-loaded control or RED system on the skin fixed in the diffusion cells. Regardless of RED system application, the vitamin C deposition was significantly higher in the stratum corneum than in the epidermis/dermis. Notably, the vitamin C deposition in the epidermis/dermis significantly increased with the application of RED system, while that in the stratum corneum was comparable between the RED and control groups.

### Effects of RED system on *in vitro* skin permeation and *in vivo* pharmacokinetics of lopinavir

**Figure 4.**
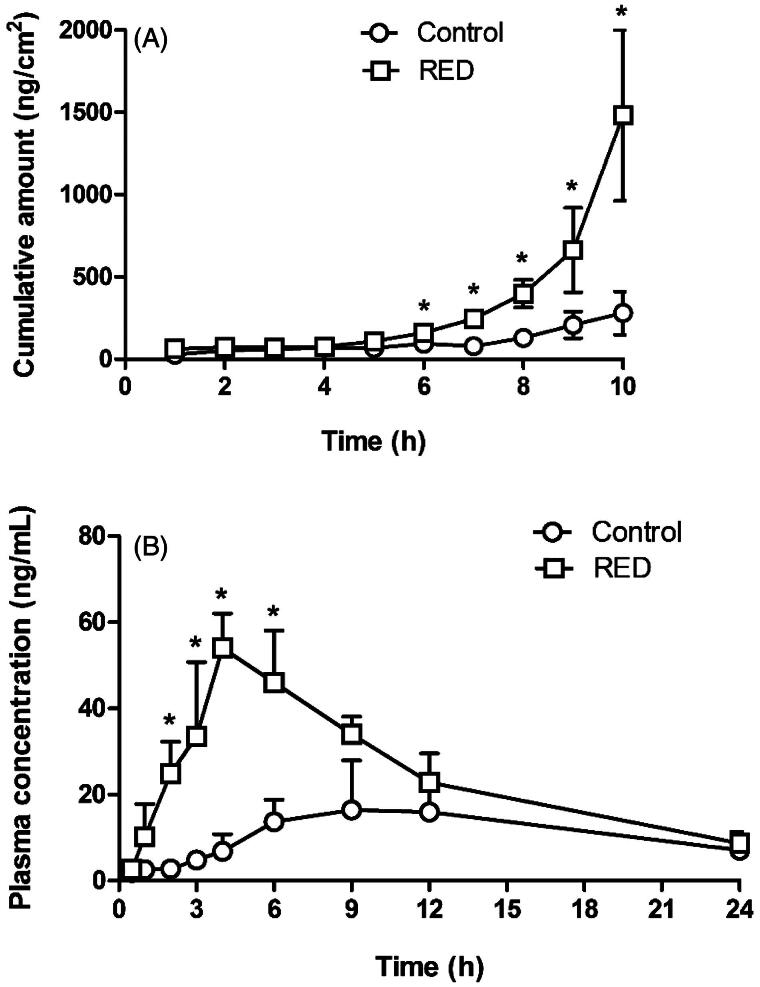
*In vitro* hairless mouse skin permeation profiles of lopinavir after the application of lopinavir-soaked gauze dressing without the RED system (control) and lopinavir-loaded RED system (RED) on the mouse skin fixed in the diffusion cells (A) and the arterial plasma concentration *versus* time profiles of lopinavir after the transdermal application of lopinavir-soaked gauze dressing without the RED system (control) and lopinavir-loaded RED system (RED) in rats (B). Bullet symbols and their error bars represent the means and standard deviations (*n* = 3–4). The asterisk (*) represents a value of the RED group significantly different from that of the control group (*p* < .05).

Figure 4(A) shows the *in vitro* hairless mouse skin permeation profiles of lopinavir after the application of lopinavir-loaded control or RED system on the skin surface. The relevant skin permeation parameters are listed in Table 2. However, as shown in Figure 4(A), no discernible linear portion was observed in the terminal phases of the plotted curves. Thus, the flux and lag time were not determined in the present lopinavir study. The *Q*_10h_ values for lopinavir were significantly higher by 5.25-fold in the RED group (99.5% of initial dose) than in the control group (19.0% of initial dose). Figure 4(B) shows the plasma pharmacokinetic profiles of lopinavir after the transdermal application of lopinavir-loaded control or RED system in rats. The relevant pharmacokinetic parameters are listed in Table 3. After the application of the RED system, plasma levels of lopinavir increased more rapidly and reached a peak (*C*_max_) at 4 h (*T*_max_), which was significantly earlier than that in the control group. The *C*_max_, *C*_ss_, and AUC_last_ values of lopinavir were also significantly higher in the RED group (2.68-, 2.21-, and 2.21-folds, respectively) than in the control group.

**Table 2.** *In vitro* skin permeation parameters of lopinavir after the application of lopinavir-soaked gauze dressing without the RED system (control) and lopinavir-loaded RED system (RED) on the hairless mouse skin fixed in the diffusion cells (*n* = 3–4).

Parameter	Control	RED
Flux (μg/h/cm^2^)	ND^b^	ND
*Q*_10 h_^a^ (μg/cm^2^)	280 ± 132	1470 ± 534*
Lag time (h)	ND	ND

^a^ Cumulative amount permeated over 10 h.

^b^ Not determined.

* Significantly different from the control group (*p* < .05).

**Table 3.** Pharmacokinetic parameters of lopinavir after the transdermal application of lopinavir-soaked gauze dressing without the RED system (control) and lopinavir-loaded RED system (RED) in rats (*n* = 3–4).

Parameter	Control	RED
*C*_max_ (μg/mL)	20.2 ± 8.7	54.1 ± 8.0*
*T*_max_ (h)	9 (6–12)	4 (4)*
*C*_ss_ (μg/mL)	11.1 ± 5.0	24.5 ± 5.4*
AUC_last_ (μg·h/mL)	267 ± 119	589 ± 129*

* Significantly different from the control group (*p* < .05).

### Effects of RED system on *in vitro* skin permeation and *in vivo* pharmacokinetics of ketoprofen

**Figure 5.**
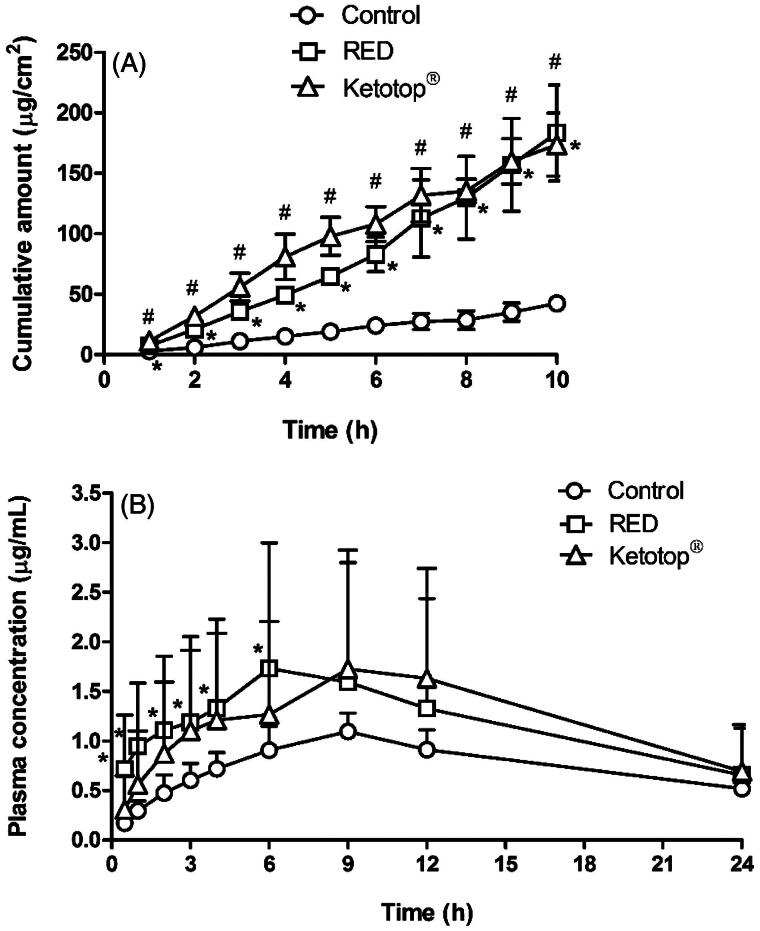
*In vitro* hairless mouse skin permeation profiles of ketoprofen after the application of ketoprofen-soaked gauze dressing without the RED system (control), ketoprofen-loaded RED system (RED), and Ketotop^®^ patch without the RED system (Ketotop^®^) on the mouse skin fixed in the diffusion cells (A) and the arterial plasma concentration versus time profiles of ketoprofen after the transdermal application of ketoprofen-soaked gauze dressing without the RED system (control), ketoprofen-loaded RED system (RED), and Ketotop^®^ patch without the RED system (Ketotop^®^) in rats (B). Bullet symbols and their error bars represent the means and standard deviations (*n* = 3–4). The asterisk (*) represents a value of the RED group significantly different from that of the control group (*p* < .05), and the pound sign (#) represents a value of the Ketotop^®^ group significantly different from that of the control group (*p* < .05).

Figure 5(A) shows the *in vitro* hairless mouse skin permeation profiles of ketoprofen after the application of ketoprofen-loaded control, RED system, or Ketotop^®^ on the skin surface. The relevant skin permeation parameters are listed in Table 4. For comparison, Ketotop^®^, a commercial ketoprofen patch formulation, was applied on the hairless mouse skin without the RED system. The flux of ketoprofen at steady state and *Q*_10 h_ were significantly higher in the RED group (by 4.68- and 4.32-folds, respectively) than in the control group. The slopes through the linear portions were readily determined by linear regression analysis with the coefficients of determination (*r*^2^) of more than 0.95. The Q_10 h_ values for the control, RED, and Ketotop^®^ groups were equivalent to 4.83%, 20.9%, and 19.8% of initial dose. These values of RED and Ketotop^®^ were also significantly higher (4.24- and 4.10-folds, respectively) than the control group. However, the lag time of ketoprofen among the three groups was not significantly different. Figure 5(B) shows the plasma pharmacokinetic profiles of ketoprofen after the transdermal application of ketoprofen-loaded control, RED system, or Ketotop^®^ in rats. The relevant pharmacokinetic parameters are listed in Table 5. The application of RED system resulted in significantly higher *C*_max_, *C*_ss_, and AUC_last_ values (2.20-, 2.10-, and 2.10-folds, respectively) than the control group. Moreover, these values were comparably high with the Ketotop^®^ group, except the lag time.

**Table 4.** *In vitro* skin permeation parameters of ketoprofen after the application of ketoprofen-soaked gauze dressing without the RED system (control), ketoprofen-loaded RED system (RED), and Ketotop^®^ patch without the RED system (Ketotop^®^) on the hairless mouse skin fixed in the diffusion cells (*n* = 3–4).

Parameter	Control	RED	Ketotop^®^
Flux (μg/h/cm^2^)	4.17 ± 0.62	19.5 ± 4.9*	17.7 ± 1.9*
*Q*_10 h_^a^ (μg/cm^2^)	42.4 ± 5.2	183 ± 40*	174 ± 26*
Lag time (h)	0.459 ± 0.371	1.14 ± 0.18	0.375 ± 0.255

^a^ Cumulative amount permeated over 10 h.

* Significantly different from the control group (*p* < .05).

**Table 5.** Pharmacokinetic parameters of ketoprofen after the transdermal application of ketoprofen-soaked gauze dressing without the RED system (control), ketoprofen-loaded RED system (RED), and Ketotop^®^ patch without the RED system (Ketotop^®^) in rats (*n* = 3–4).

Parameter	Control	RED	Ketotop^®^
*C*_max_ (μg/mL)	1.16 ± 0.13	2.55 ± 0.72*	2.32 ± 0.43*
*T*_max_ (h)	9 (9–12)	6 (6–9)	9 (9–12)
*C*_ss_ (μg/mL)	0.748 ± 0.074	1.57 ± 0.36*	1.61 ± 0.24*
AUC_last_ (μg·h/mL)	18.0 ± 1.77	37.8 ± 8.7*	38.8 ± 5.8*

* Significantly different from the control group (*p* < .05).

### *In vivo* dermal toxicity study in rats

**Figure 6.**
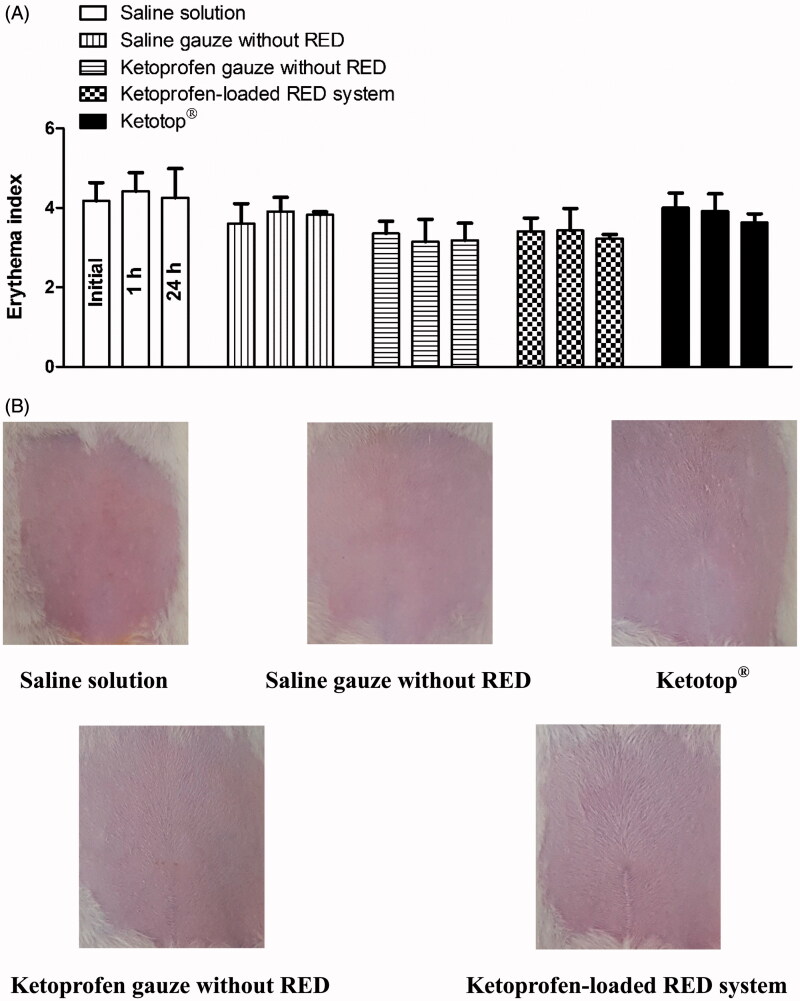
Erythema indices of skin at 0, 1, and 24 h (A) and its photographs at 24 h (B) after the transdermal application of saline solution, saline-soaked gauze dressing without the RED system (Saline gauze without RED), ketoprofen-soaked gauze dressing without the RED system (Ketoprofen gauze without RED), ketoprofen-loaded RED system, and Ketotop^®^ patch without the RED system (Ketotop^®^) in rats. The rectangular bars and their error bars represent the means and standard deviations (*n* = 3–4).

Skin irritation caused by the developed RED system was assessed using the rat skin erythema test. Figure 6(A) shows the erythema indices at 0, 1, and 24 h after the transdermal application of saline solution, saline-soaked gauze dressing, ketoprofen-loaded gauze dressing and RED system, and Ketotop^®^ patch in rats. No significant difference in the erythema indices was observed at the three time points among all the experimental groups tested (Figure 6(A)). Images of rats at 1 h (Figure S7) and 24 h (Figure 6(B)) also demonstrate that no discernible changes in skin were observed during the entire period of the toxicity study.

## Discussion

The present study provides novel data on the application of our newly developed RED system for the topical delivery of FITC-HA and vitamin C and the transdermal delivery of lopinavir and ketoprofen in hairless mouse skin and Sprague–Dawley rat models. As our RED system is intended for dermal use, it needs to be sufficiently small and lightweight, and hence, the size of the RED battery was fixed at eight pairs of cells. In our previous study, after the electrical reaction was initiated by adding brine to the RED battery, the electric current produced from the RED system increased rapidly, reaching a peak within a few minutes, and then decreased slowly over 1 h (Lee et al., 2017). Moreover, in a preliminary test, we measured the current density produced from the RED system applied on the hairless mouse and human skin (*n* = 3 for each group). The maximal current density values observed in three mice ranged from 372 to 575 μA/cm^2^ and in three humans ranged from 379 to 459 μA/cm^2^. This result implies that the electrical performance of the RED system is comparable to that of conventional iontophoretic systems (typically <500 μA/cm^2^) (Calatayud-Pascual et al., 2011). Therefore, we attempted to apply the RED system for topical and/or transdermal delivery of therapeutic agents whose dermal applications have been limited due to their physicochemical properties.

Our previous study reported the cross-sectional CLSM images of FITC-PLL distribution within the porcine skin (Lee et al., 2017). However, in the present study, the CLSM analysis was performed in the Z-stack image-generating mode with intact hairless mouse skin. Thus, we performed the Z-stack CLSM analysis with FITC-PLL to confirm the validity of the modified experimental conditions related to the CLSM analysis. As shown in Figure 2, the fluorescence density of FITC-PLL at the skin depths of 12 and 30 μm (corresponding to the epidermis/dermis) was significantly enhanced by the RED system, which shows similar results to those reported in our previous study (Lee et al., 2017). Furthermore, the CLSM analysis with FITC-HA showed that the fluorescence density of FITC-HA at a skin depth of 3 and 12 μm (corresponding to the stratum corneum and epidermis/dermis, respectively) was significantly enhanced by the RED system (Figure 2). This result clearly indicates that the RED system significantly enhanced the permeation of FITC-HA across the stratum corneum and its deposition in the epidermis/dermis. Similarly, as shown in Figure 3, the RED system significantly enhanced the deposition of vitamin C in the epidermis/dermis. As the epidermis/dermis is considered as the primary site of action of topically administered HA and vitamin C, it is plausible that the current RED system can enhance their biological and pharmacological effects on the skin. Moreover, the *in vitro* skin permeation parameter (*Q*_10_) and *in vivo* pharmacokinetic parameters (*C*_ss_ and AUC) of lopinavir significantly increased when the RED system was applied (Tables 2 and 3), suggesting that the RED system successfully enhanced the skin permeation and systemic absorption of transdermally administered lopinavir.

It is well known that iontophoretic system enhances molecular transport across the skin through two main mechanisms: electrorepulsion and electroosmosis (Calatayud-Pascual et al., 2011). Electrorepulsion, also known as electromigration, refers to the ordered movement of ions through the skin by direct interaction with the applied electric field (Kalia et al., 2004). In this mechanism, the repulsion between a charged molecule and electrode of the same polarity acts as a driving force for the permeation of the charged molecule through the skin. As vitamin C and HA exist primarily in the form of anion at the pH of skin, it is plausible that the application of RED system enhanced the topical delivery of the two substances by electrorepulsion. Meanwhile, skin can be viewed as a negatively charged membrane at physiological pH. When an electrical current is applied across the skin, the net flow of solvent can occur in the direction of movement of the counter ions with positive charge, which is referred to as electroosmosis (Calatayud-Pascual et al., 2011). The electroosmotic flow of solvent can carry any solutes in the anode-to-cathode direction though the skin, acting as an additional driving force, particularly for the permeation of neutral and large-sized molecules (Guy et al., 2001). As lopinavir exists as a neutral molecule at the pH of skin, it is suggested that the RED system enhanced its transdermal delivery by electroosmosis. However, further investigation is required to clarify the exact mechanism of the effects of the RED system on the topical and transdermal delivery of the therapeutic agents observed in this study.

The efficiency of transdermal delivery and skin irritation was compared between the RED system and Ketotop^®^ patch, a commercially available transdermal patch formulation of ketoprofen. As shown in Tables 4 and 5, the extent of increases in the *in vitro* skin permeation parameters (flux and *Q*_10_) and *in vivo* pharmacokinetic parameters (*C*_ss_ and AUC) of ketoprofen were comparable between the two groups. Moreover, the results shown in Figures 6 and S7 indicate that no skin irritation was observed after applying either. These results suggest that the application of RED system can offer transdermal delivery efficiency and safety profiles equivalent to those of the commercial available Ketotop^®^ patch. Furthermore, we expect that the RED system can be readily developed as a potable, compact, and disposable patch formulation, which can be a good alternative to conventional iontophoresis systems.

## Conclusion

The present study demonstrated that our novel RED-driven iontophoretic system significantly enhanced the efficiency of topical and transdermal delivery of HA, vitamin C, and lopinavir. Moreover, the transdermal delivery efficiency and safety of the RED system were shown to be comparable with those of the commercial ketoprofen patch formulation. To the best of our knowledge, this is the first study on the utility of the RED-driven iontophoretic system for topical and transdermal drug delivery. We believe that the RED system can serve as an efficient topical and transdermal delivery system for various poorly bioavailable therapeutic agents, including HA, vitamin C, and lopinavir, in the near future.

## Supplementary Material

IDRD_Kim_et_al_Supplemental_Content.zip
