# Supplementary material for: Novel reverse electrodialysis-driven iontophoretic system for topical and transdermal delivery of poorly permeable therapeutic agents
Source: Drug Deliv. 2017 Aug 28;24(1):1204–15. doi: 10.1080/10717544.2017.1367975 (PMC8241169; doi:10.1080/10717544.2017.1367975)
Supplement: IDRD_Kim_et_al_Supplemental_Content.zip [file IDRD_A_1367975_SM8997.zip › Supplementary Information-DDEL-R1.docx]

***Supplementary Information***

**Novel reverse electrodialysis-driven iontophoretic system for topical and transdermal delivery of poorly permeable therapeutic agents**

Ki-Taek Kim^1*^, Joon Lee^2*^, Min-Hwan Kim^1^, Ju-Hwan Park^1^, Jae-Young Lee^3^, Joo-Hyun Song^2^, Minwoong Jung^2^, Myoung-Hoon Jang^2^, Hyun-Jong Cho^4^, In-Soo Yoon^5^, and Dae-Duk Kim^1^

*^1^College of Pharmacy and Research Institute of Pharmaceutical Sciences, Seoul National University, Gwanak-gu, Seoul, Republic of Korea, ^2^Biosensor Laboratories Inc., Seoul National University, Gwanak-gu, Seoul, Republic of Korea, ^3^College of Pharmacy, Chungnam National University, Yuseong-gu, Daejeon, Republic of Korea, ^4^College of Pharmacy, Kangwon National University, Chuncheon-si, Gangwon, Republic of Korea, and ^5^College of Pharmacy, Pusan National University, Geumjeong-gu, Busan, Republic of Korea*

^*^These authors contributed equally to this work.

Address for correspondence: In-Soo Yoon, College of Pharmacy, Pusan National University, 2 Busandaehak-ro 63beon-gil, Geumjeong-gu, Busan 46241, Republic of Korea. E-mail: insoo.yoon@pusan.ac.kr; Dae-Duk Kim, College of Pharmacy and Research Institute of Pharmaceutical Sciences, Seoul National University, 1 Gwanak-ro, Gwanak-gu, Seoul 08826, Republic of Korea. E-mail: ddkim@snu.ac.kr

Video S1. Please see the attached video file to visualize the electric current generated from the RED system. Sense wires (5806, Keithley Instruments, INC., Cleveland, OH, USA) were connected to the electrodes of the RED battery and a bulb. After adding diluted NaCl solution to the upper side of the RED battery, the bulb lit up. The intensity of bulb light decreased slowly over time as the charge gradient between the concentrated layer and diluted layer decreased.


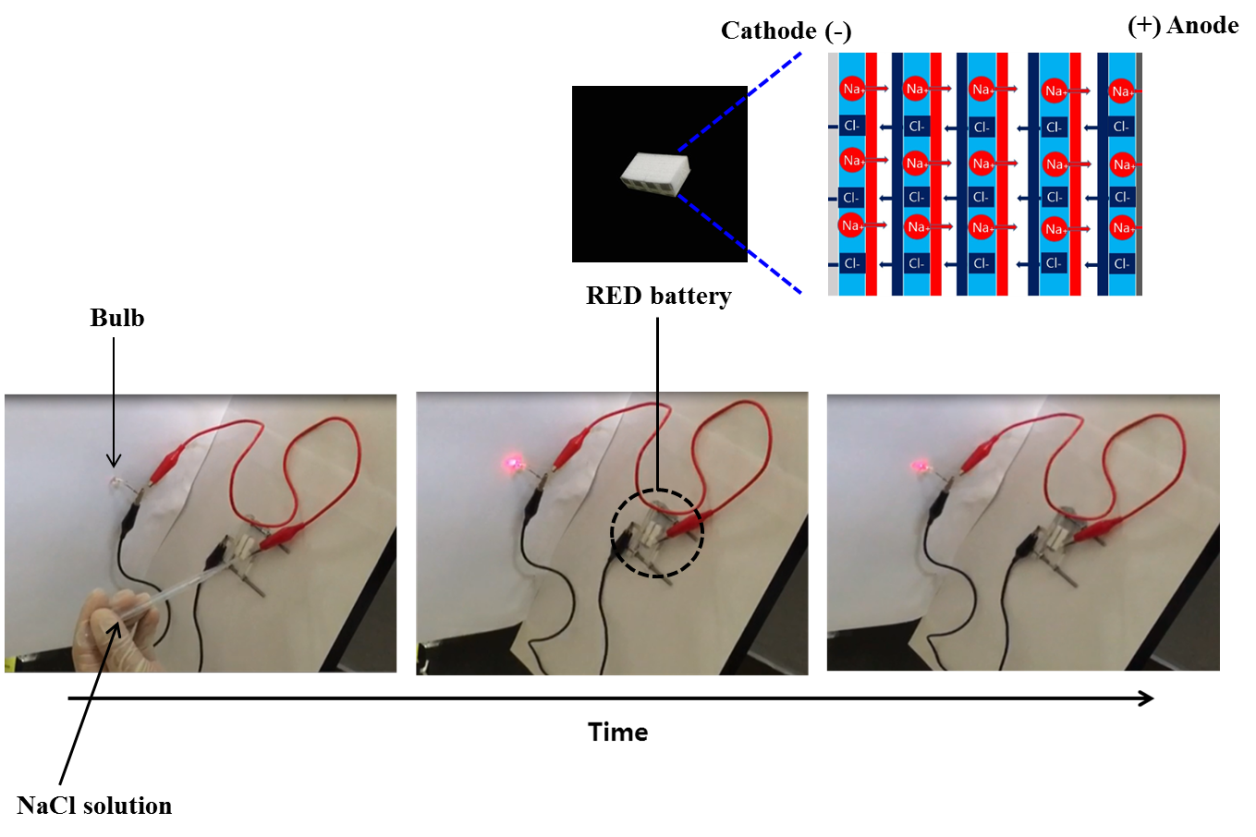


Figure S1. The bulb lighting after adding NaCl solution to the RED system.

A


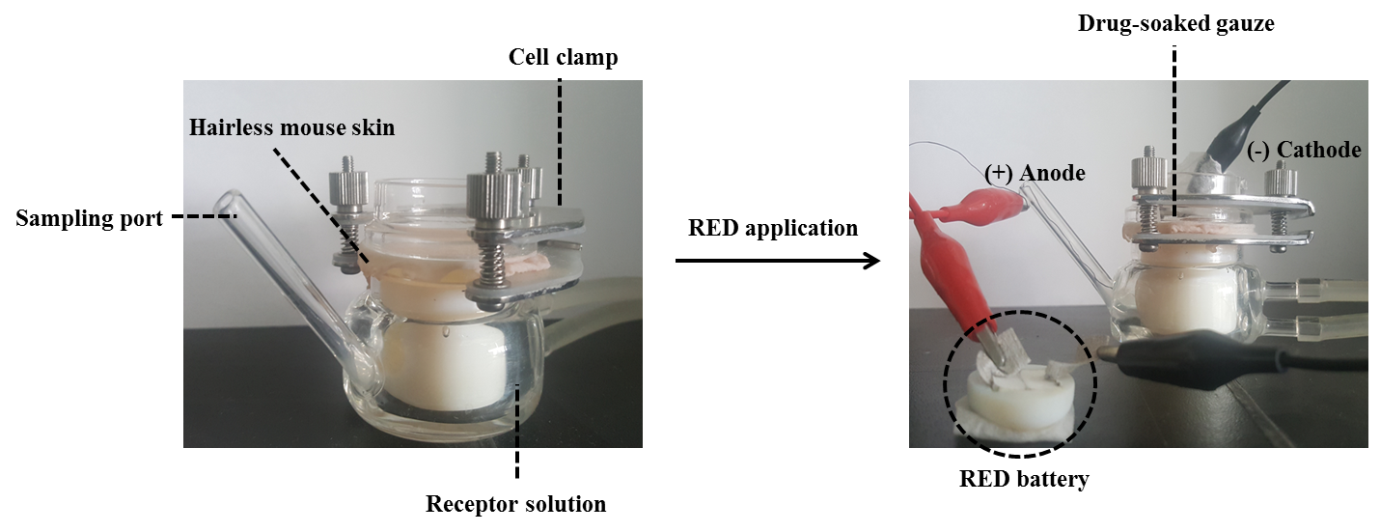


B


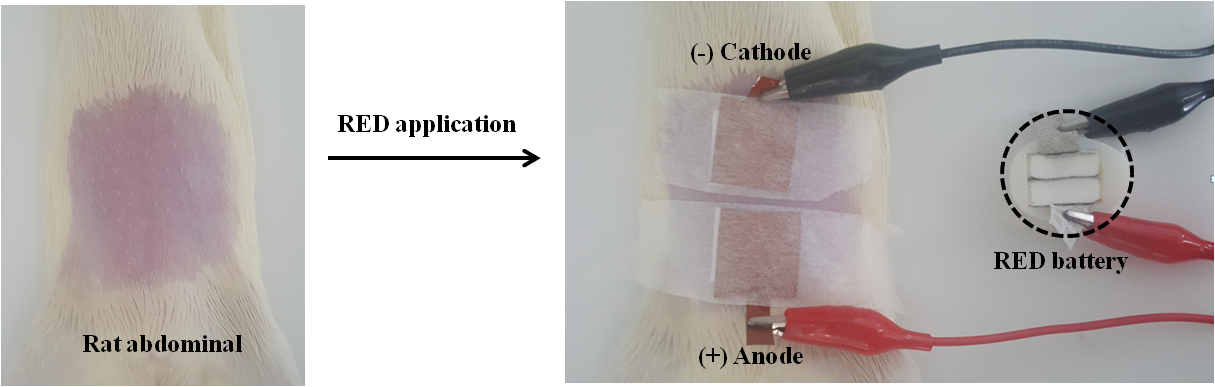


Figure S2. Images of Keshary–Chien diffusion cells set up for *in vitro* skin permeation study (A) and rats fixed on a plate for the *in vivo* pharmacokinetic study using the RED system (B).

| **A** | **B** |
| --- | --- |
| 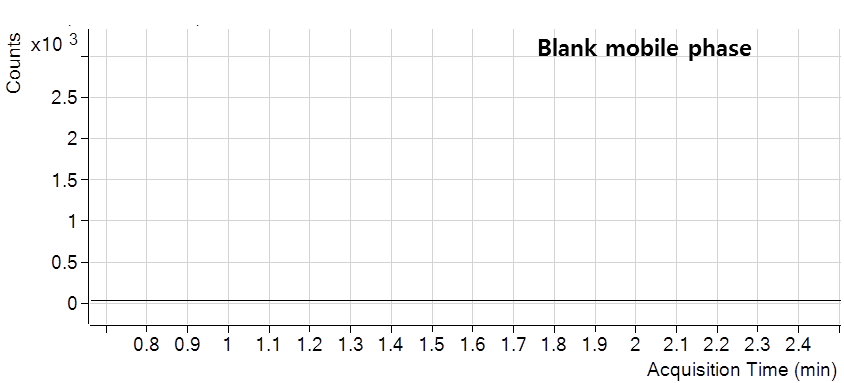 | 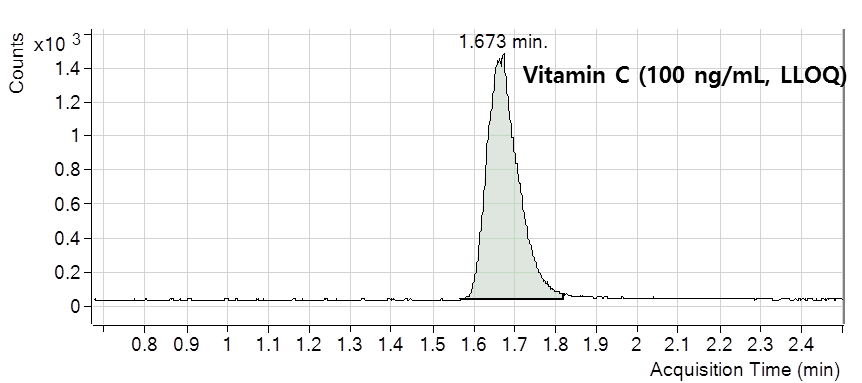 |
|  |  |
| **C** | **D** |
| 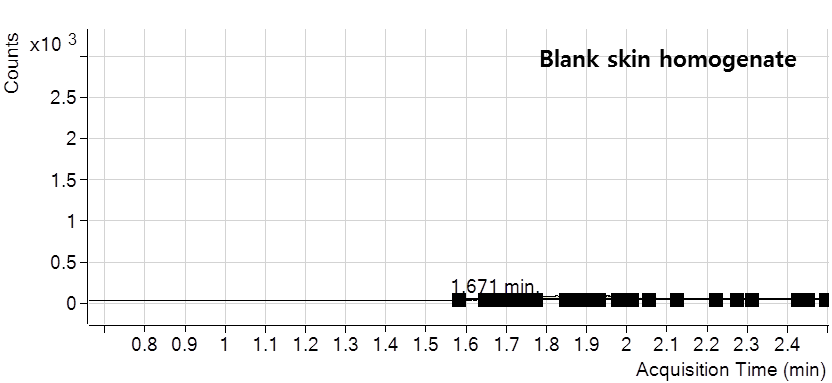 | 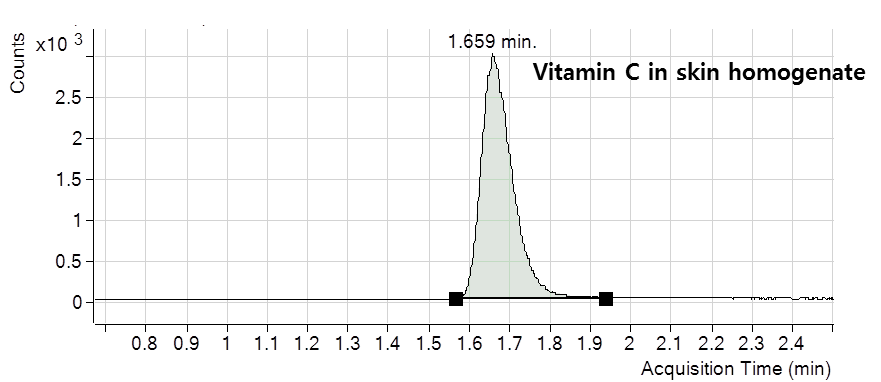 |

Figure S3. Representative LC-MS/MS chromatograms of vitamin C in blank mobile phase (A), vitamin C standard solution at a concentration of 100 ng/mL, LLOQ (B), blank hairless mouse skin homogenate samples (C), and hairless mouse skin homogenate samples obtained in the *in vitro* skin deposition study of vitamin C (D).

| **A** | **B** |
| --- | --- |
| **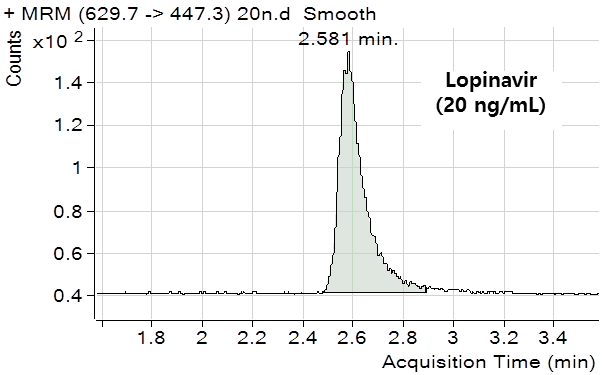** | **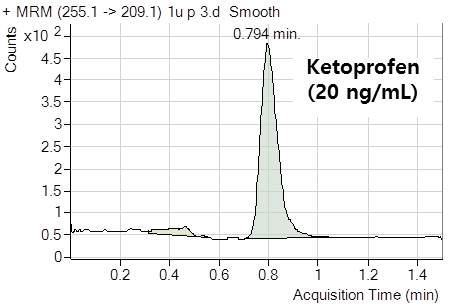** |
|  |  |
| **C** | **D** |
| **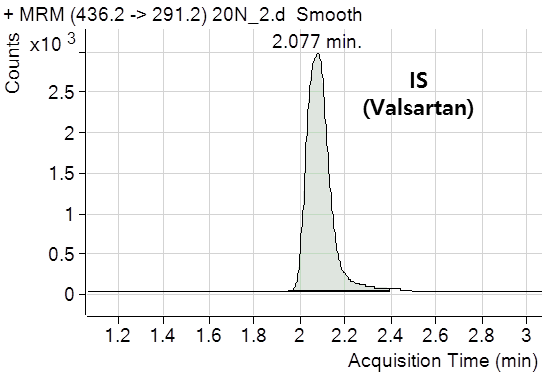** | **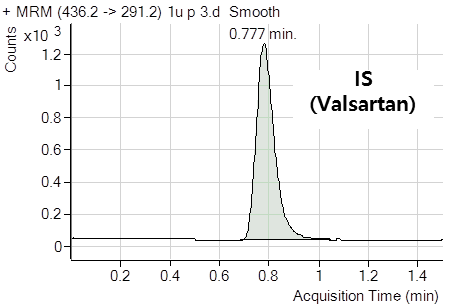** |

Figure S4. Representative LC-MS/MS chromatograms of lopinavir (A) and ketoprofen (B) and their internal standard (IS, valsartan) (C and D) in the rat plasma samples spiked with lopinavir (5 ng/mL, LLOQ) or ketoprofen (20 ng/mL, LLOQ) and IS (375 ng/mL).

A

| **Skin depth**  **(μm)** | **Control** | | **RED** | |
| --- | --- | --- | --- | --- |
|  | **1 hr** | **6 hr** | **1 hr** | **6 hr** |
| **Z=0** | **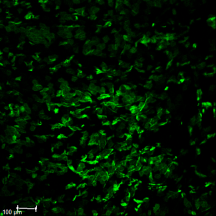** | **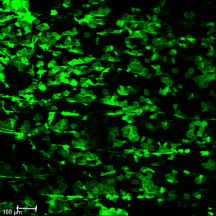** | **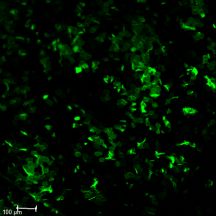** | **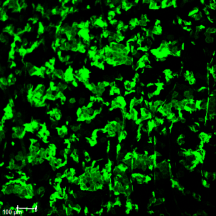** |
| **Z=3** | **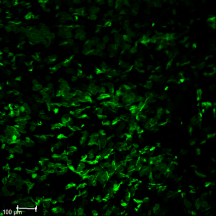** | **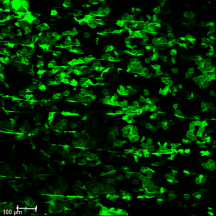** | **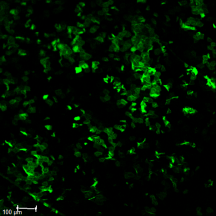** | **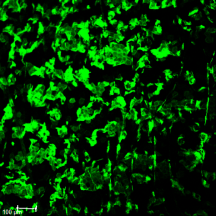** |
| **Z=6** | **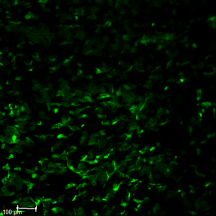** | **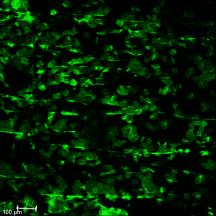** | **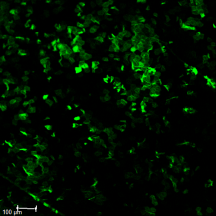** | **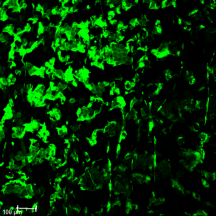** |
| **Z=9** | **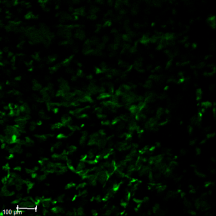** | **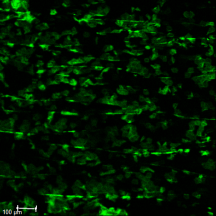** | **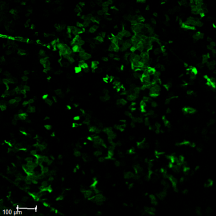** | **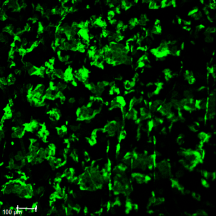** |
| **Z=12** | **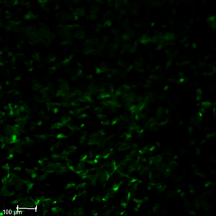** | **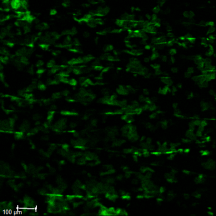** | **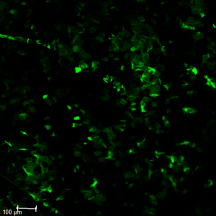** | **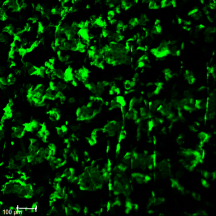** |
| **Z=15** | **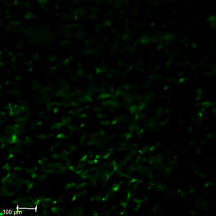** | **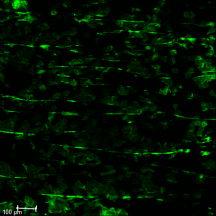** | **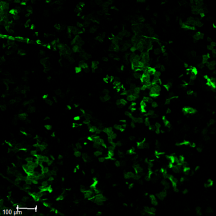** | **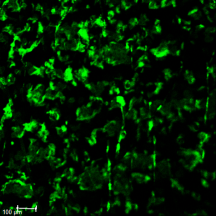** |
| **Z=18** | **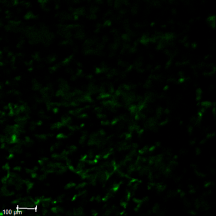** | 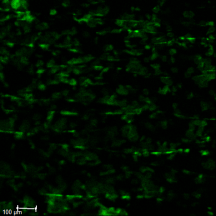 | **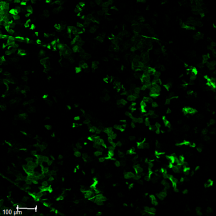** | **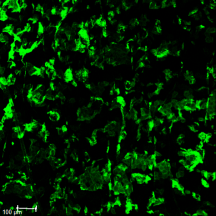** |
| **Z=21** | **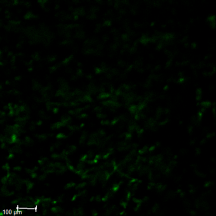** | **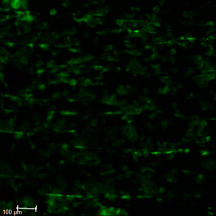** | **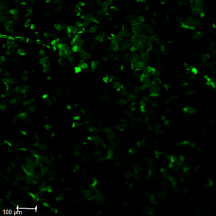** | **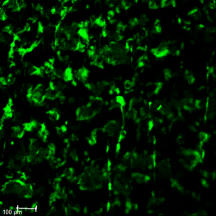** |
| **Z=24** | **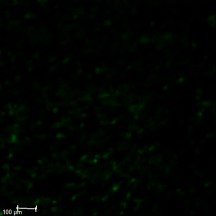** | **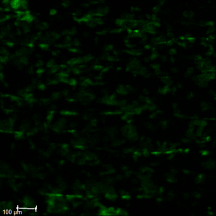** | **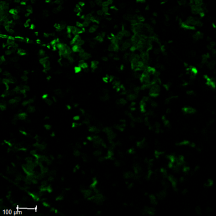** | **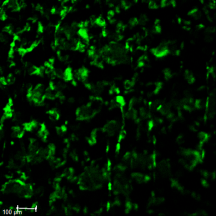** |
| **Z=27** | **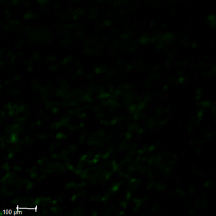** | **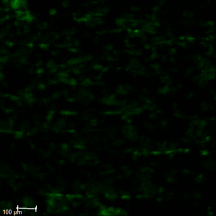** | **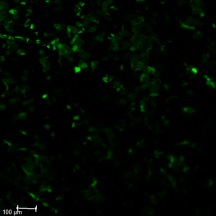** | **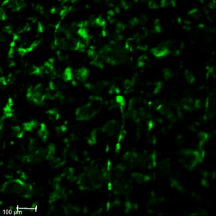** |
| **Z=30** | **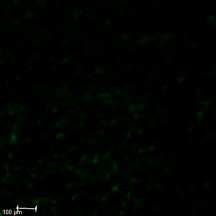** | 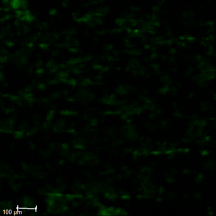 | **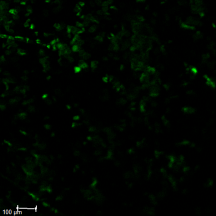** | **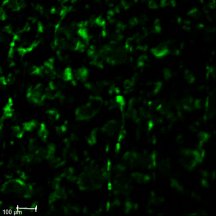** |

**B**

| **Skin depth**  **(μm)** | **Control** | | **RED** | |
| --- | --- | --- | --- | --- |
|  | **1 hr** | **6 hr** | **1 hr** | **6 hr** |
| **Z=0** | **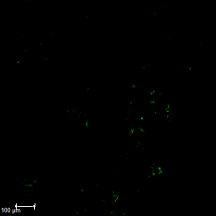** | **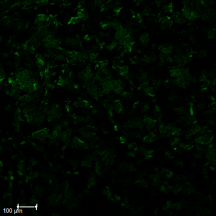** | **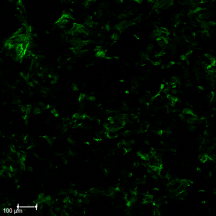** | **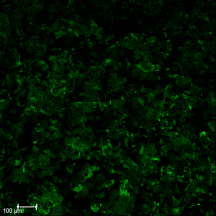** |
| **Z=3** | 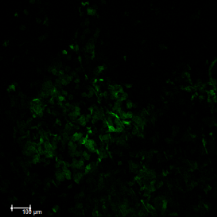 | 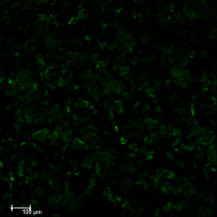 | 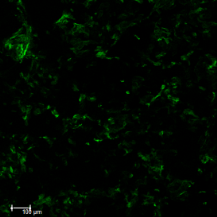 | 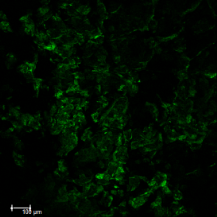 |
| **Z=6** | **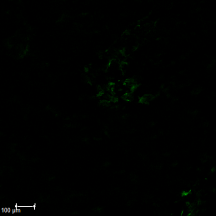** | **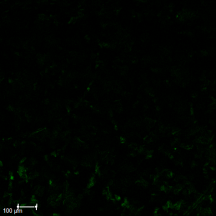** | **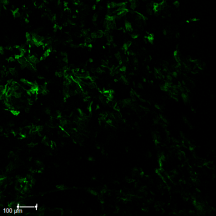** | **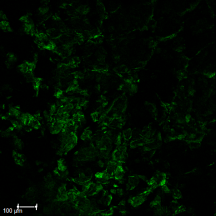** |
| **Z=9** | **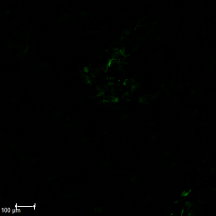** | **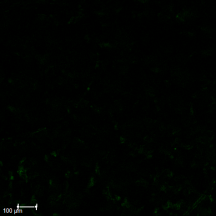** | **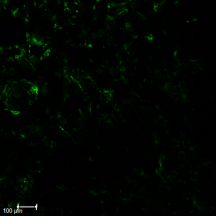** | **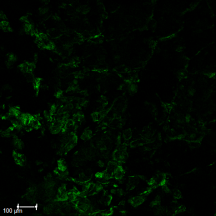** |
| **Z=12** | 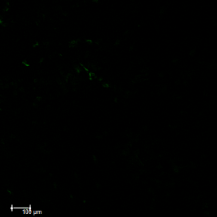 | 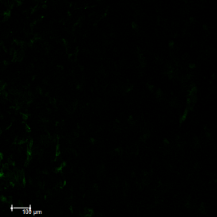 | 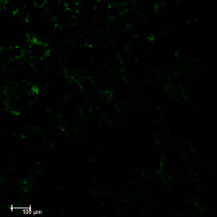 | 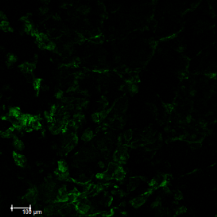 |
| **Z=15** | **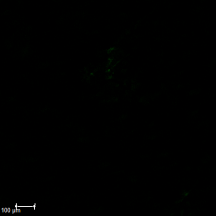** | **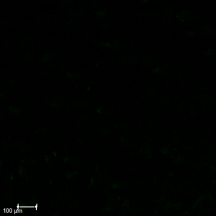** | **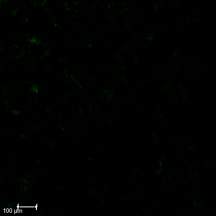** | **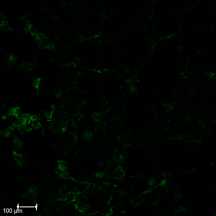** |
| **Z=18** | **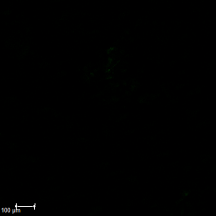** | 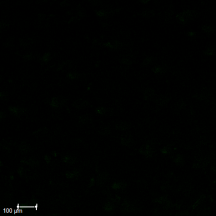 | **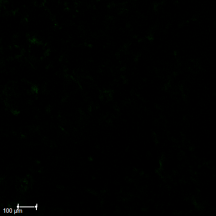** | **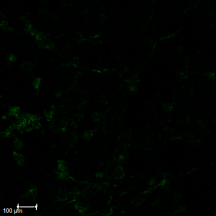** |

Figure S5. Z-stack CLSM images for FITC-PLL (A) and FITC-HA (B) at stepwise-increased skin depths (3 μm interval) of hairless mouse skin at 1 h and 6 h after the application of FITC-PLL- or FITC-HA-soaked gauze dressing without the RED system (control) and FITC-PLL- or FITC-HA-loaded RED system (RED) on the mouse skin fixed in the diffusion cells. The fluorescence signal emitted from FITC-PLL or FITC-HA was represented by green color. The scale bars represent 100 μm.

**A**

|  | **X/Z penetration** | **X/Y/Z penetration** |
| --- | --- | --- |
| **Control**  **at 1 h** | **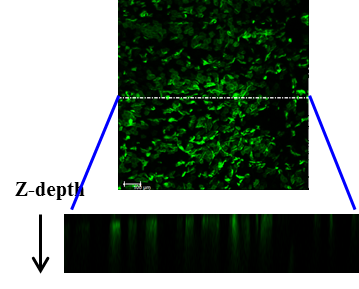** | **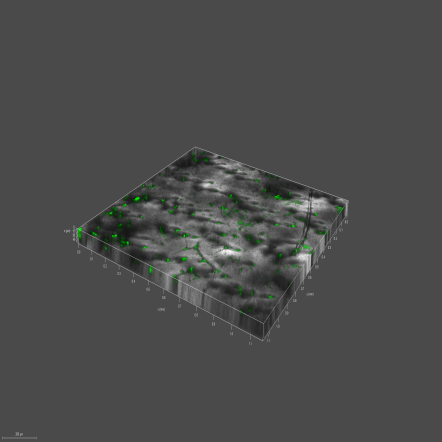** |
| **Control**  **at 6 h** | **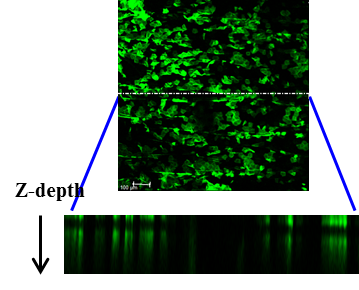** | **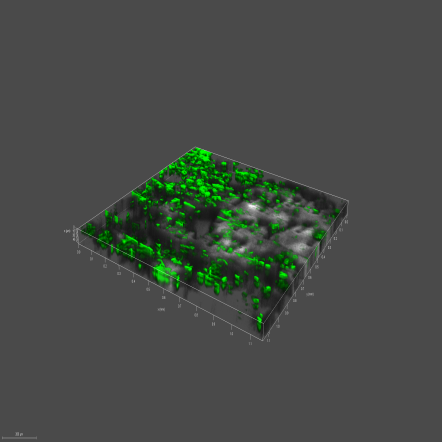** |
| **RED**  **at 1 h** | **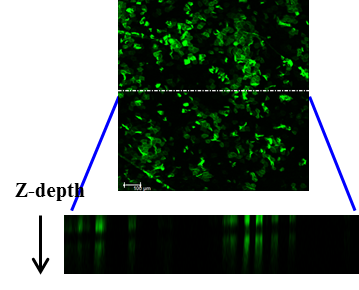** | **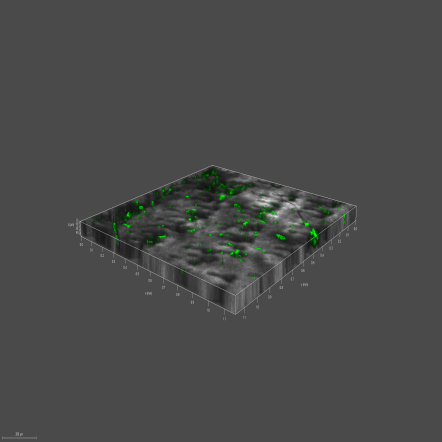** |
| **RED**  **at 6 h** | 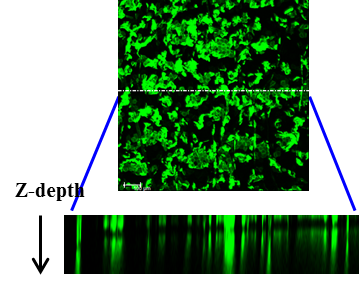 | **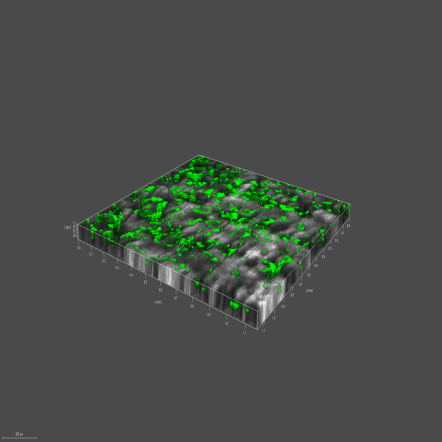** |

**B**

|  | **X/Z penetration** | **X/Y/Z penetration** |
| --- | --- | --- |
| **Control**  **at 1 h** | **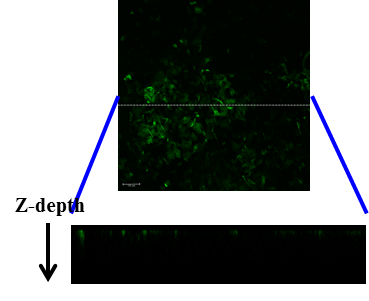** | **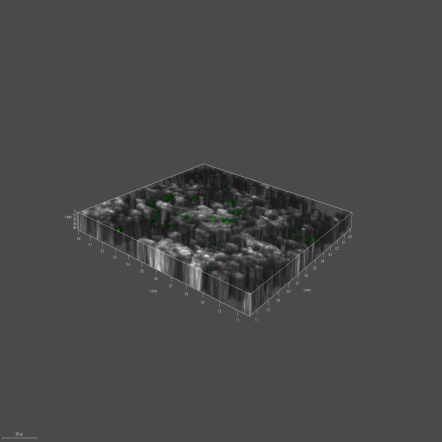** |
| **Control**  **at 6 h** | **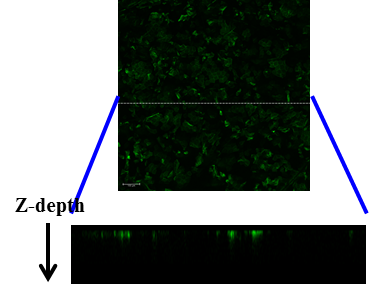** | **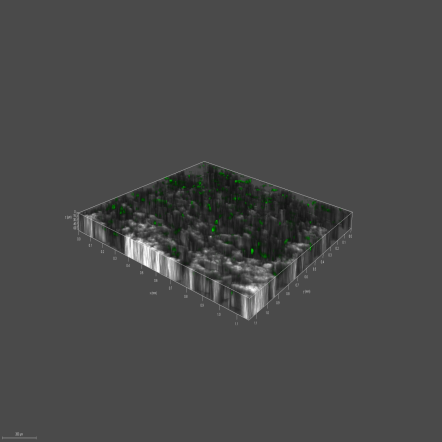** |
| **RED**  **at 1 h** | **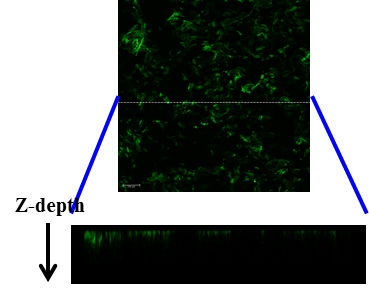** | **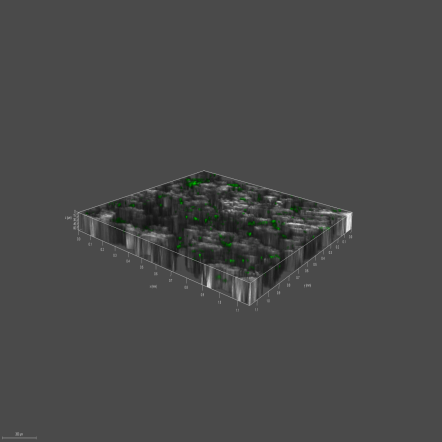** |
| **RED**  **at 6 h** | 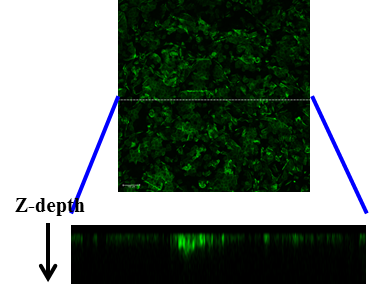 | **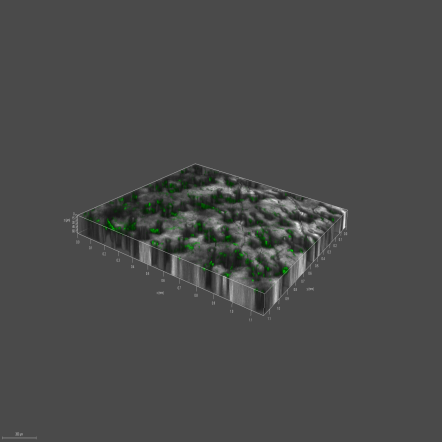** |

Figure S6. X/Z-penetration and X/Y/Z-penetration (3 dimension) CLSM images for FITC-PLL (A) and FITC-HA (B) at all skin depths (3 μm interval) of hairless mouse skin at 1 h and 6 h after the application of FITC-PLL- or FITC-HA-soaked gauze dressing without the RED system (control) and FITC-PLL- or FITC-HA-loaded RED system (RED) on the mouse skin fixed in the diffusion cells. The fluorescence signal emitted from FITC-PLL or FITC-HA was represented by green color.

|  | **Saline solution** | **Saline gauze without RED** | **Ketoprofen gauze without RED** | **Ketoprofen-loaded**  **RED system** | **Ketotop^®^** |
| --- | --- | --- | --- | --- | --- |
| **Before**  **the application** | 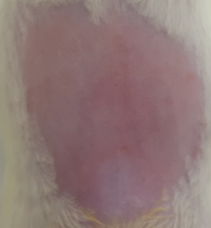 |  |  |  |  |
| **At 1 h after**  **the application** |  |  |  |  |  |

Figure S7. Images of abdominal skin before the application and at 1 h after the transdermal application of saline solution, saline-soaked gauze dressing without the RED system (Saline gauze without RED), ketoprofen-soaked gauze dressing without the RED system (Ketoprofen gauze without RED), ketoprofen-loaded RED system, and Ketotop^®^ patch without the RED system (Ketotop^®^) in rats.
